# Improving photosynthesis through multidisciplinary efforts: The next frontier of photosynthesis research

**DOI:** 10.3389/fpls.2022.967203

**Published:** 2022-09-30

**Authors:** Xin-Guang Zhu, Mirza Hasanuzzaman, Anjana Jajoo, Tracy Lawson, Rongcheng Lin, Chun-Ming Liu, Lu-Ning Liu, Zhenfeng Liu, Congming Lu, Michael Moustakas, Thomas Roach, Qingfeng Song, Xinyou Yin, Wangfeng Zhang

**Affiliations:** ^1^Center of Excellence for Molecular Plant Sciences, Chinese Academy of Sciences, Shanghai, China; ^2^Department of Agronomy, Faculty of Agriculture, Sher-e-Bangla Agricultural University, Dhaka, Bangladesh; ^3^School of Biotechnology, Devi Ahilya University, Indore, India; ^4^School of Life Science, University of Essex, Colchester, United Kingdom; ^5^Key Laboratory of Photobiology, Institute of Botany, Chinese Academy of Sciences, Beijing, China; ^6^School of Advanced Agricultural Sciences, Peking University, Beijing, China; ^7^Institute of Systems, Molecular and Integrative Biology, University of Liverpool, Liverpool, United Kingdom; ^8^National Laboratory of Biomacromolecules, Institute of Biophysics, Chinese Academy of Sciences, Beijing, China; ^9^School of Life Sciences, Shandong Agricultural University, Taian, China; ^10^Department of Botany, School of Biology, Aristotle University of Thessaloniki, Thessaloniki, Greece; ^11^Department of Botany, University of Innsbruck, Innsbruck, Austria; ^12^Center of Excellence for Molecular Plant Sciences, Chinese Academy of Sciences, Shanghai, China; ^13^Department of Plant Sciences, Centre for Crop Systems Analysis, Wageningen University & Research, Wageningen, Netherlands; ^14^Department of Agronomy, Shihezi University, Shihezi, China

**Keywords:** photosynthesis, photobiology, multiscale, efficiency, modeling, natural variation, synthetic biology

The light-dependent release of oxygen from plants was first discovered in the 1770s by Joseph Priestley and Jan Ingenhousz. More recently, the enzyme-catalyzed pathway of carbon assimilation was characterized by Melvin Calvin, James Bassham, and Andrew Benson in 1950, and since then, photosynthesis has been intensively studied by hundreds and thousands of other pioneers. So far, the major components of photosynthesis in different systems and the regulations over these components have been gradually revealed. Now, photosynthesis research is entering a new era, with the ambitious goal of providing new green solutions for overcoming the challenges facing our society, such as ensuring the sustainable supply of food, fiber, and fuel, as well as improving the ecological stability of our planet. We can also conceive that one day we may also leave our planet to live on others, but certainly not without photoautotrophs! Developing photosynthetic systems, both natural and artificial, with greater efficiency in using resources, such as light, nitrogen, CO_2_, and water, to benefit human society and our planet, is becoming a new frontier of research and a hallmark of this exciting era of photosynthesis research.

## Why is there large scope to improve photosynthesis?

There are great variations in the photosynthetic energy conversion efficiency in extant plants, which are nonetheless usually less than 1/3 of the theoretical optimal photosynthetic light use efficiency (Zhu et al., 2008; Slattery and Ort, 2015; Yin and Struik, 2015). The increased production of biomass and yield in major crops under Free Air CO_2_ Enrichment experiments shows that increasing photosynthesis can indeed increase crop yield (Long et al., 2006). Many arguments can be used to explain why evolution has not resulted in optimal photosynthesis. First, evolution selects for survival over productivity. Usually, one particular anatomical, physiological or developmental feature, which confers better tolerance to a particular stress, can offer plants higher fitness in their growth habitat regardless of whether the plant by chance has a high photosynthetic rate or not. As an extreme example, having mechanisms to maintain a high water use efficiency, e.g., the Crassulacean acid metabolism, will be a preferred option for survival in an extremely dry environment while maintaining a superior photosynthetic rate becomes less critical under this condition. Second, since photosynthesis first evolved, there have been dramatic changes in climate, such as CO_2_ levels, temperature, precipitation, etc., all historically leading to specialized adaptations that can now be considered “outdated.” Just in the past 200 years, atmospheric CO_2_ levels increased from about 200 ppm to 410 ppm, average global temperatures have soared by 1.5°C and precipitation has become increasingly erratic (IPCC Climate Change, 2021). Such rapid changes open up possibilities to optimize photosynthesis toward current and future climate scenarios. Furthermore, the climate is changing at a speed faster than the speed of plant adaptation. This has been demonstrated earlier, for example, by the kinetic properties of Rubisco, for which kinetic properties fit better to the CO_2_ level of 400,000 years ago (Zhu et al., 2004). The changes in the global temperature also have a major impact on photosynthetic performance (Sage and Kubien, 2007). Thus, sub-optimality of photosynthesis can be related to the legacy of evolution. Rubisco evolved 2.4 billion years ago, which was a hypoxic and CO_2_-rich environment, under which the inefficiency of Rubisco carboxylation activity was not relevant (Banda et al., 2020).

In contrast to these reasons why evolution has not selected the optimal photosynthesis, there are also other parallel arguments on the current photosynthetic properties that may already represent an “optimal” choice for plants. The balance between plant growth and stress resistance in a highly variable and potentially stressful environment may also prevent the maximization of photosynthesis and hence growth potential (Zhang et al., 2020), i.e., the diverse photosynthetic properties in nature represent different evolutionary choices for plants to survive and thrive in their habitats without human intervention. Along this vein, it is interesting to note that, green plants, purple bacteria, and green sulfur bacteria have drastically different absorption spectra, however, their current light absorption spectrum may be an “optimal” design for the light conditions they commonly experience (Arp and Kistner-Morris, 2020). Similarly, Rubisco, being able to catalyze both ribulose bisphosphate (RuBP) carboxylation and also RuBP oxygenation, may also well represent an evolutionary preferred choice compared to a hypothetically perfect Rubisco, which can only catalyze RuBP carboxylation. This is again because, under stress conditions, this RuBP oxygenation capacity can not only help dissipate excess light energy but also help maintain a metabolite pool which can, when needed, be used to rapidly provide intermediates for the Calvin-Benson cycle (Stitt and Borghi, 2021).

A few factors underlie huge opportunities to improve photosynthesis. First, the rapid global climate change outpaced the speed of plant evolution, as discussed earlier. Second, during crop domestication, crops have drastically different growth habitats compared to those of their ancestors. For example, modern crops usually are grown in monoculture as a dense canopy, as compared to their ancestors which often have access to plenty of sunlight. Thirdly, compared to the situation of plants growing in the wild, which can only rely on their repertoire of weapons and solutions to cope with stresses, crops in agriculture can be protected through human intervention (irrigation, fertilizer application, disease control, etc). As a result, plants can take a competitive growth strategy, rather than a stress tolerant or ruderal strategy (Grime, 1977).

Though it is desirable for plants to have a high demand for improving photosynthetic efficiency, it is worth noting here that, under certain conditions, such as under high light, the photosynthetic efficiency becomes less important, while effective photoprotective mechanisms and ensuring total photosynthetic yield becomes more relevant for plants. Indeed, sophisticated mechanisms have evolved to ensure high photosynthetic yield under high light, especially under concurrent high light and stress combinations, while at the same time confer higher quantum yield when the light becomes a scarce resource (Ort, 2001). This scenario again implies opportunities to utilize the excess energy which is otherwise largely wasted in photoprotection, as shown in the recent success of engineering a faster recovery from photoprotection for greater biomass production (Kromdijk et al., 2016).

Here, we emphasize that the rapid development of synthetic biology tools now offers new opportunities to create completely new designs of improved photosynthetic systems and tailoring photosynthesis to the increasing demands in the context of our changing climate (Zhu et al., 2020). In the following sections, we briefly discuss the available opportunities, the tools used to support studying photosynthesis, and the associated research areas.

## An incomplete list of options to improve photosynthesis

First, we provide an incomplete list of opportunities to improve photosynthetic efficiency:

(1) Creating more efficient light harvesting systems, which could utilize the expanded solar spectrum for the generation of proton motive force for generation of ATP and NADPH (Ort et al., 2015) and/or smaller chlorophyll antenna size and lower chlorophyll content reducing the excess absorption of sunlight and improving photosynthetic efficiency (Ort et al., 2011; Moustakas et al., 2022).
(2) Creating more efficient photo-protection systems to minimize heat dissipation when unnecessary and to maximize photochemistry;
(3) Creating more efficient state transition and electron transferring between PSII and PSI under changing environment to maximize light-use efficiency;
(4) Generating a Rubisco with a greater catalytic rate and higher specificity for CO_2_;
(5) Repurposing efficient CO_2_-concentrating mechanisms, either these are Kranz type CO_2_-concentrating mechanisms, or carboxysome- or pyrenoid-based systems to decrease the Rubisco oxygenation flux;
(6) Creating a novel pathway to cope with photorespiratory CO_2_ and ammonia loss to minimize the energy associated with refixation of CO_2_ and ammonia;
(7) Developing an effective combination of biological CO_2_ fixation with solar energy capture to further increase the efficiency of harvesting light energy through capitalizing on the high light conversion efficiency of photovoltaic systems;
(8) Developing nanomaterials to enable better capturing and delivery of CO_2_ to Rubisco to decrease the Rubisco oxygenation;
(9) Enhancing antioxidant defense under natural changing conditions to decrease the photodamage;
(10) Development of artificial systems that cope better with high light different conditions through channeling the excess light for production of renewable chemical energy;
(11) Overcoming sink limitations of photosynthesis;
(12) Developing improved stomatal dynamics to increase water and light use efficiency.
(13) Develop photosynthetic systems that can better utilize fluctuating light conditions;
(14) Creation of novel photosynthetic systems which may enable human space exploration.

These are all basic elements required to build a repertoire of highly efficient systems. When these modules for higher efficiency are individually developed, or achieved in combination, we could gain increased plant yield potential either for biomass or grain or storage tissues, as well as greener energy sources.

## Technologies and tools to support a new era of photosynthesis research

The rapid progress in many new technologies and tools provides sufficient toolsets for us to overcome these grand challenges and goals (Table 1). These major technological advances that will revolutionize photosynthesis research in the future include:

- **Techniques to scan photosynthetic systems**. The advances in the fluorescence imaging techniques for *in vivo* scanning of natural photosynthetic systems will enable *in situ* characterization of photosynthetic pigment-protein complexes and their distribution/dynamics under different conditions (Casella et al., 2017; Mullineaux and Liu, 2020). This information and elucidation of their physiological significance will provide basic information which is needed for the future de novo design of artificial photosynthetic systems.
- **Techniques for studying the molecular and supramolecular basis of photosynthesis**. The recent development of cryo-electron microscopy (cryo-EM) technology enables rapid progress in solving the structures of major proteins, protein complexes and supercomplexes involved in photosynthesis at near-atomic resolutions through the single-particle analysis method (Wei et al., 2016; Su et al., 2017; Zhang et al., 2017; Malone et al., 2019; Pi et al., 2019; Pan et al., 2020). Atomic force microscopy (AFM) technology provides the opportunity to delineate the lateral arrangement, protein interactions and dynamics of photosynthetic complexes in the context of photosynthetic membranes (Liu and Scheuring, 2013; Wood et al., 2018; Zhao et al., 2020). In addition, the cryo-electron tomography (cryo-ET) method allows researchers to visualize the *in situ* arrangement of photosynthetic complexes in chloroplasts and CO_2_-fixing organelles (Engel et al., 2015; Freeman Rosenzweig et al., 2017; Wietrzynski et al., 2020; Gupta et al., 2021; Ni et al., 2022). The structural information combined with the variation of genomic sequences and corresponding changes in biophysical or biochemical properties of the proteins/protein complexes will enable the determination of the molecular and supramolecular basis underlying photosynthetic processes and regulation. The detailed physical mechanisms can then be revealed through a combination of such biological or genetic manipulation, e.g., base editing, experiments with molecular dynamics simulations, especially those that combine quantum mechanics and molecular mechanics (MoD QM/MM) (Liguori et al., 2020) and artificial intelligence-based protein structure prediction (Jumper et al., 2021). It is worth pointing out here that the combination of multiple approaches will not only enable studies on the structure-function relationship of proteins or protein complexes, but also may stimulate *ab initio* design of new protein/protein complexes with desired properties (Hsia et al., 2021).
- **Techniques to mine superiority within the natural variation of photosynthesis**. Though in current plants, a photosynthetic system with all components in an optimal state is not yet available, there are great natural variations of photosynthetic machinery across diverse photosynthetic organisms. High-throughput plant phenotyping techniques combined with genome-wide association techniques and genetic tools can allow the identification of novel genes controlling photosynthetic efficiency and characterize those genetic variations conferring superior traits for some components of photosynthesis.
- **Multi-scale systems modeling of photosynthesis**. The modeling will allow not only the dissection of biological, biophysical, and biochemical mechanisms controlling the efficiency of a particular photosynthetic protein or complex, but also the rational design of optimal photosynthetic systems for greater efficiency under different environments (Xiao et al., 2017). Novel photosynthetic models enable accurate prediction of the photosynthesis process and its regulation at different scales still needs to be developed.
- **Availability of versatile synthetic biology tools allowing targeted manipulation of photosynthesis**. A highly efficient photosystem requires a strongly coordinated expression of genes that encode the photosystem components. Many promoters that confer precise temporal, spatial, or tissue-specific expression of target genes are available (Kummari et al., 2020); furthermore, with rapid advances in single-cell transcriptomics and stereomics data, new promoters conferring either temporal or spatial or development, or environment specificity will be rapidly identified (Xia et al., 2022). CRISPR-CAS9 tools enable fine-tuning expression levels and translation efficiency have been developed (Jiang and Doudna, 2017). All these will enable an unprecedented opportunity to design and implement new photosynthetic systems.
- **Guided evolution techniques**. Though during evolution, photosynthetic efficiency might not be a target that selection acts on, we can now implement guided evolution where optimal photosynthetic efficiency or enhanced properties of specific photosynthetic proteins can be a target for the experiment, as shown in the development of new CO_2_ fixation pathway in *Escherichia coli* (Antonovsky et al., 2016). Guided evolution tools combined with artificial intelligence can be used to support the optimization of photosynthetic proteins, such as Rubisco or new photosynthetic pathways, or even the creation of new options for improved photosynthetic efficiency, taking advantage of the power of random mutation and selection.

**Table 1:** Methods used to study photosynthesis at different scales.

	**Protein, pigment protein complexes and thylakoid membrane scale**	**Chloroplast, cellular and leaf scales**	**Cellular and leaf scales**
Tools for structure and morphological characterization	Cryo-EM, cryo-ET, AFM, transmission EM, freeze-fracture EM, fluorescence imaging	Cryo-ET, Scanning EM, light microscope	Image based phenomics
Signals used for functional characterization	Fluorescence emission, absorption spectrum, gas exchange signal, oxygen evolution, isotope discrimination, metabolomics, fluxomics	Gas exchange signal, fluorescence emission signal, reflectance signal	Gas exchange signal, fluorescence emission signal, reflectance signal
Theoretical models used to describe photosynthesis (or a component of photosynthesis) at different scales	Molecular dynamics models, quantum mechanics models, molecular mechanics models, reaction diffusion models	Reaction diffusion models, dynamic systems models, steady state models	Steady state models, dynamic systems models, reaction diffusion models

## Research areas on photosynthesis to support development of new strategies for improved photosynthetic efficiency

As the basic process ultimately responsible for the generation of food, fiber, and fuel for our society, and also a crucial component of the global carbon cycle and the water cycle, photosynthesis is arguably one of the most important biological processes on this planet. Understanding how photosynthesis works, and how to further optimize it, will be a never-ending pursuit of humanity. Photosynthesis not only supplies materials and energy supporting our living organisms on Earth, but also holds the promise to provide the basic needs for humans in the forthcoming era of space life. The Photosynthesis and Photobiology section of Frontiers in Plant Sciences provides a unique arena for scientists working in this field to publish their recent advances in these fields; it will also be a window for industrial partners and stakeholders to present their recent development. Advances in photosynthesis research will be a showcase of the triumph of multi-disciplinary research across the diversity of photosynthetic organisms. This Photosynthesis and Photobiology section will welcome high-quality fundamental and applied research across all areas of photosynthesis and photobiology, which include but are not limited to:

- Architecture, assembly, biogenesis, and functional regulation of pigment-protein complexes, supercomplexes, and megacomplexes involved in the light reactions.
- Structure and mechanism of enzymes and transporters associated with photosynthesis and their regulation
- Structure and function of thylakoid membrane systems
- Mechanisms of light energy absorption, transfer, and conversion processes under different light regimes
- Structure, function, genetics, and reconstruction of different CO_2_-concentrating mechanisms (CCM)
- Structure and variation of gene regulatory networks controlling photosynthesis
- Factors controlling stomatal conductance and mesophyll conductance
- Factors controlling leaf and canopy photosynthesis
- Structure, function and genetic regulation of crassulacean acid metabolism
- Photosynthesis under changing climate conditions or stress conditions
- Photosynthesis under different supplies of either macromineral or microelement
- Multiscale models of photosynthesis
- Evolution of photosynthesis
- Photosynthesis on planets other than earth
- Natural variation of photosynthesis and their genetic basis
- Synthetic biology of photosynthesis for better enzymes, systems, or pathways
- Crop improvement for higher photosynthetic efficiency under a changing climate
- Light-induced signal transduction and photomorphogenesis
- Artificial photosynthesis and clean energy generation
- Creation of new hybrid photosynthetic systems for greater light use efficiency.

**Figure 1:**
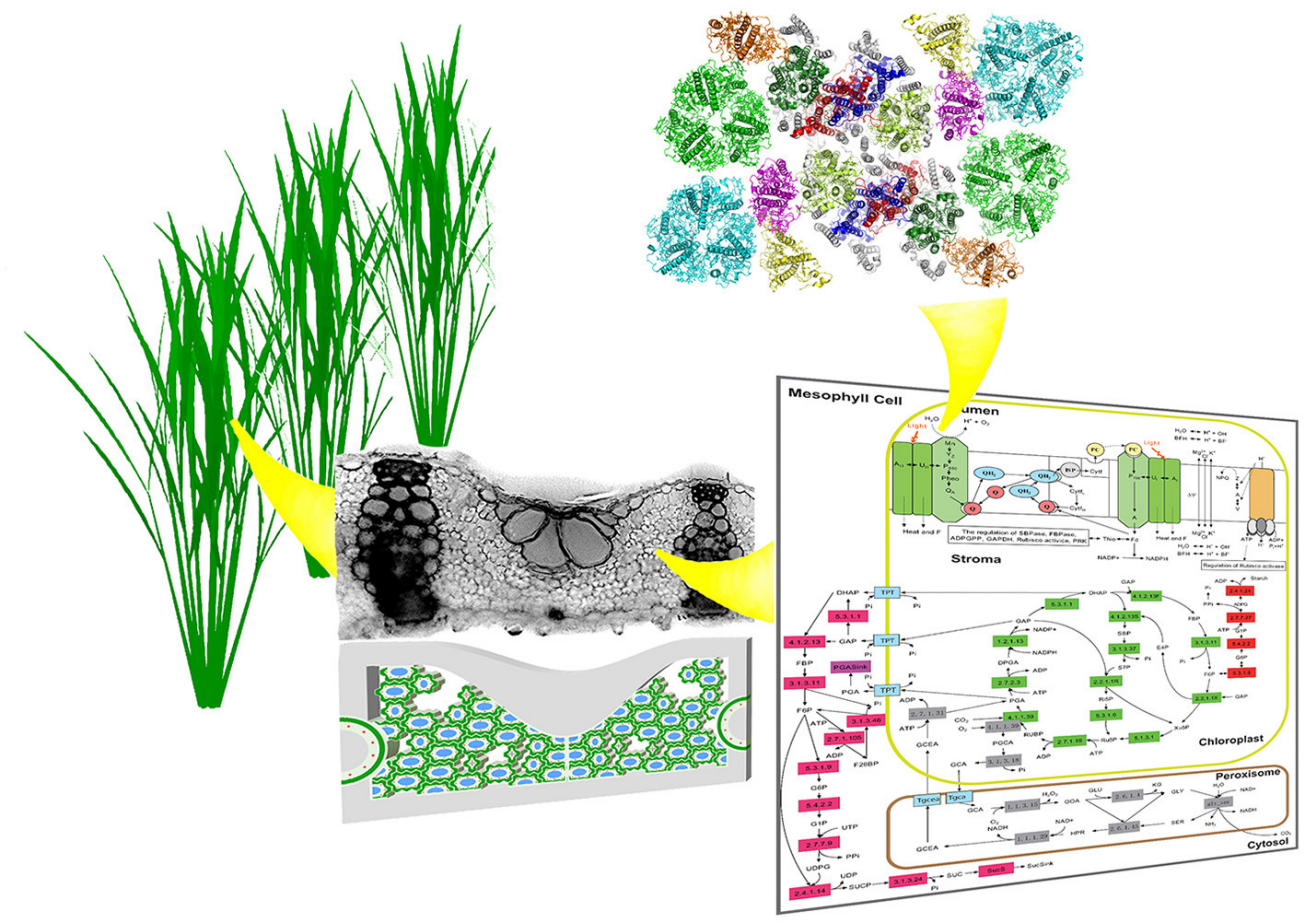
Photosynthesis research involves research at different scales. Here we illustrate the multi-scale properties of photosynthesis with the higher plant systems. Systematic studies of the structure, function and regulation of photosyhnthesis at canopy, plant, leaf, cell, chloroplast, pigment protein complexes *etc* are needed to develop effective methods to identify factors controlling photosynthetic efficiency and hence to design effective approaches to improve photosynthetic efficiency.

Photosynthesis research is entering a new era, where more and more work targets at enhancing its efficiency, in addition to the characterization of natural photosynthetic systems. Given that efficiency is inherently a system's property, i.e., it is a result of all the interacting components, rather than any single component. As a result, studying photosynthetic efficiency and identifying new options to improve efficiency will inevitably require the examination of photosynthesis at a range of spatial and temporal scales (Figure 1). Therefore, in this new era of photosynthesis research, we will witness the final success of capitalizing on the power of photosynthesis tailored to gain optimal efficiency for different environments, which will rely on accurate *in silico* prediction of photosynthesis in action from the first principle based on the spatial arrangement of photosynthetic pigment-protein complexes, and sequence and structure of individual proteins involved. After centuries of research on photosynthesis, the twenty-first century will witness how photosynthesis research will help advance our agricultural and energy development, and sustainably maintain or even improve our environment. The Photosynthesis and Photobiology section of Frontiers in Plant Sciences will serve as an arena for the whole photosynthesis research community to team up and work together to welcome this new era of photosynthesis research.

## Author contributions

X-GZ drafted the article. All authors contributed to the article and approved the submitted version.

## Conflict of interest

The authors declare that the research was conducted in the absence of any commercial or financial relationships that could be construed as a potential conflict of interest.

## Publisher's note

All claims expressed in this article are solely those of the authors and do not necessarily represent those of their affiliated organizations, or those of the publisher, the editors and the reviewers. Any product that may be evaluated in this article, or claim that may be made by its manufacturer, is not guaranteed or endorsed by the publisher.
